# Safety and Efficacy of an Injectable Solution Enriched With Sodium Hyaluronate, Amino Acids, and Peptides in Relation to Superficial Facial Connective Tissues (Dermis and Retinacular Cutis)

**DOI:** 10.1111/jocd.16586

**Published:** 2024-09-15

**Authors:** Evgeniya Shelemba, Olha Olshanska, Anne Gaelle Benoit, Elena Rumyantseva

**Affiliations:** ^1^ P.L. Shypuk National Medical Academy of Post‐Graduate Education Kyiv Ukraine; ^2^ Innovaesthetic Kyiv Ukraine; ^3^ Private Practice London UK; ^4^ Private Practice Paris France

**Keywords:** amino acids, collagen, connective tissue, facial ligaments, HA, peptides, retinacular cutis

## Abstract

**Background:**

Skin aging research often focuses on the dermis, overlooking the significance of the retinacular cutis (RC) in aging. This study aimed to investigate the efficacy, safety, and effect of an injectable solution containing hyaluronic acid, amino acids, and peptides, on facial sagging and laxity by targeting the RC.

**Methods:**

This single‐center observational study recruited 28 female volunteers aged 25–65 years. The participants received four monthly injections of the studied solution. Objective measures included skin hydration, elasticity, color, thickness, collagen density evaluated via DermaLab Combo and high‐resolution ultrasound imaging. Subjective measures included participant satisfaction evaluated using the Global Aesthetic Improvement Scale (GAIS). Adverse effects were monitored throughout the study period.

**Results:**

Significant improvements were observed in skin hydration, elasticity, and collagen density after treatment. Hydration increased by 25.9% at T1 (30 days after last session), sustaining a 15.9% increase at T2 (120 days after last session). Elasticity improved by 29.2% at T1 and 20.7% at T2. Collagen density increased by 20.27% at T1 and 16.71% at T2. Self‐reported GAIS scores showed consistent increases. Adverse effects were minimal and included only transient ecchymosis and mild pain.

**Conclusion:**

Injections of the solution had a substantial hydrating effect, enhanced elasticity, and increased collagen density in the RC and dermis. Results persisted at the 120‐day follow‐up, indicating sustained benefits. Hence, this injectable solution may offer a safe and effective non‐invasive treatment option for improving skin laxity and sagging, targeting the RC and other deep connective tissue such as retaining ligaments.

## 1 Introduction

Although research on skin aging has often focused on the dermis and its role in maintaining skin structure and firmness, recent studies have started to shed more light on the significance of the retinacular cutis in the overall aging process. The retinacular cutis, also known as the superficial fascial system or subcutaneous tissue retinacula, is a complex network of loosely interlaced collagen fibers that lies just beneath the skin. It is an extension of the connective tissue from the superficial fascia to the dermis, which connects the skin to the deeper layers of tissue, including muscles and bones, effectively suspending the skin and allowing it to move in tandem with the body's movements [1]. The retinacular cutis plays a crucial role in maintaining skin supply and elasticity. It supports the dermis, where collagen and elastin fibers are abundant, and aids in the distribution of nutrients and oxygen to various layers of the skin [2, 3]. As we age, the retinacular cutis undergoes changes similar to those of other components of the skin [4, 5]. These changes can contribute to visible signs of aging, such as wrinkles, sagging, and loss of skin firmness [6].

Recognizing the importance of this often‐overlooked component can lead to innovative rejuvenation approaches tailored to preserve the health and structure of the face effectively. Numerous studies have shown that injections of hyaluronic acid (HA), amino acids (AAs), and peptides are efficacious and well tolerated for skin quality improvement [7, 8]. However, only few studies have reported the effect of a combination of HA, AAs, and peptides on the improvement of facial connective tissues, such as the dermis and retinacular cutis. Jalupro Super Hydro contains a combination of high‐ and low‐molecular‐weight HA, AAs, and peptides and is indicated to not only improve collagen levels in the dermis but also rejuvenate the retinacular cutis, unlocking new possibilities in aesthetic medicine. This study aimed to investigate the potential benefits, safety, and efficacy of the injectable solution Jalupro Super Hydro (manufactured by Professional Derma SA). We focused on investigating the synergistic effects of a subdermal injection of the combination of low‐ and high‐molecular weight sodium hyaluronate, an amino acid cluster, and three peptides in participants with facial skin sagging and laxity. Particularly, the study assessed the impact of this combination on various aspects of the facial skin, including biophysical parameters; participant satisfaction; and potential adverse effects.

## 2 Materials and Methods

### 2.1 Study Design and Participants

This single‐center, open‐label, observational study was conducted according to the tenets of the Declaration of Helsinki. Informed consent was obtained, and all participants allowed the publication of their photographs before the initiation of the study.

This study was conducted from December 2022 to July 2023. A total of 28 healthy adult female volunteers aged 25–65 years were included. The inclusion criterion was signs of connective tissue aging (skin laxity and facial sagging). The exclusion criteria were as follows: (1) autoimmune soft tissue and neoplastic diseases; (2) ongoing anti‐inflammatory therapy; (3) inflammatory or infectious diseases affecting the treatment area; (4) presumed or confirmed sensitivity towards one of the ingredients of the product; (5) hypersensitivity to HA, amide local anesthetics, or streptococcal proteins; (6) history of bleeding disorders, porphyria, or keloids; (7) male sex, as sex‐related differences in the skin matrix influence viscoelastic properties; (8) age <25 or >65 years of age owing to age‐related differences in skin properties; (9) smoking, as it dehydrates the skin; (10) refusal to sign the photo release consent form; (11) concomitant medications or diseases that affect skin hydration or cause increased levels of swelling or bruising; and (12) herpes or other local facial infections at the time of procedure.

All participants were required to refrain from any cosmetic or surgical procedures thought to be confounding, including neurotoxin injections, peelings, facials, or energy‐based devices, in the treatment area (face) at least 3 months before the beginning, during the study, and 4 months after the end of the clinical investigation.

We evaluated the therapeutic effects of a pre‐mixed injectable product (Jalupro Super Hydro; Professional Derma SA) containing 80 mg of a sterile gel of non‐cross‐linked sodium hyaluronate (high and low molecular weight) with a specific mixture of seven AAs (Glycine, L‐lysine, L‐leucine, L‐proline, L‐ arginine, L‐alanine, L‐valine) and three peptides (acetyl‐decapeptide‐3, oligopeptide‐24, acetyl‐tetrapeptide‐5) on the dermis and retinacular cutis of the face. One apyrogenic disposable glass syringe containing 2.5 mL of gel is a medical device regulated by the Medical Device Directive 93/42/EEC.

The product was injected four times at 4‐week intervals. Subjective and objective assessments (efficacy evaluations) were recorded at T0, that is before each treatment session (baseline assessment); T1, that is, 30 days after the last treatment session (short‐term assessment to capture immediate post‐treatment changes and improvements); and T2, that is, 120 days after the last treatment session (long‐term assessment) to evaluate any delayed effects and assess the durability and longevity of the treatment effects.

### 2.2 Objective Assessments

Before the first treatment, each subsequent treatment, and 30 and 120 days after the last treatment, the investigator cleaned each participant's face with clean water and dried with soft cloth tissue, followed by rest for 30 min in a quiet room. Photographs were taken using a camera, and the degree of skin sagging was analyzed. Then, the skin properties were objectively evaluated; skin hydration (moisture), trans‐epidermal water loss (TEWL), skin color (degree of melanin and erythema), elasticity, skin thickness, and collagen density were tested with the DermaLab Combo skin analyzing device (Cortex Technology, Denmark) in a room with a constant room temperature at 24°C and relative humidity of 50%. The skin thickness and collagen density in the dermis and retinacular cutis were determined using a high‐resolution ultrasound standard probe, and ultrasound images were recorded.

**FIGURE 1:**
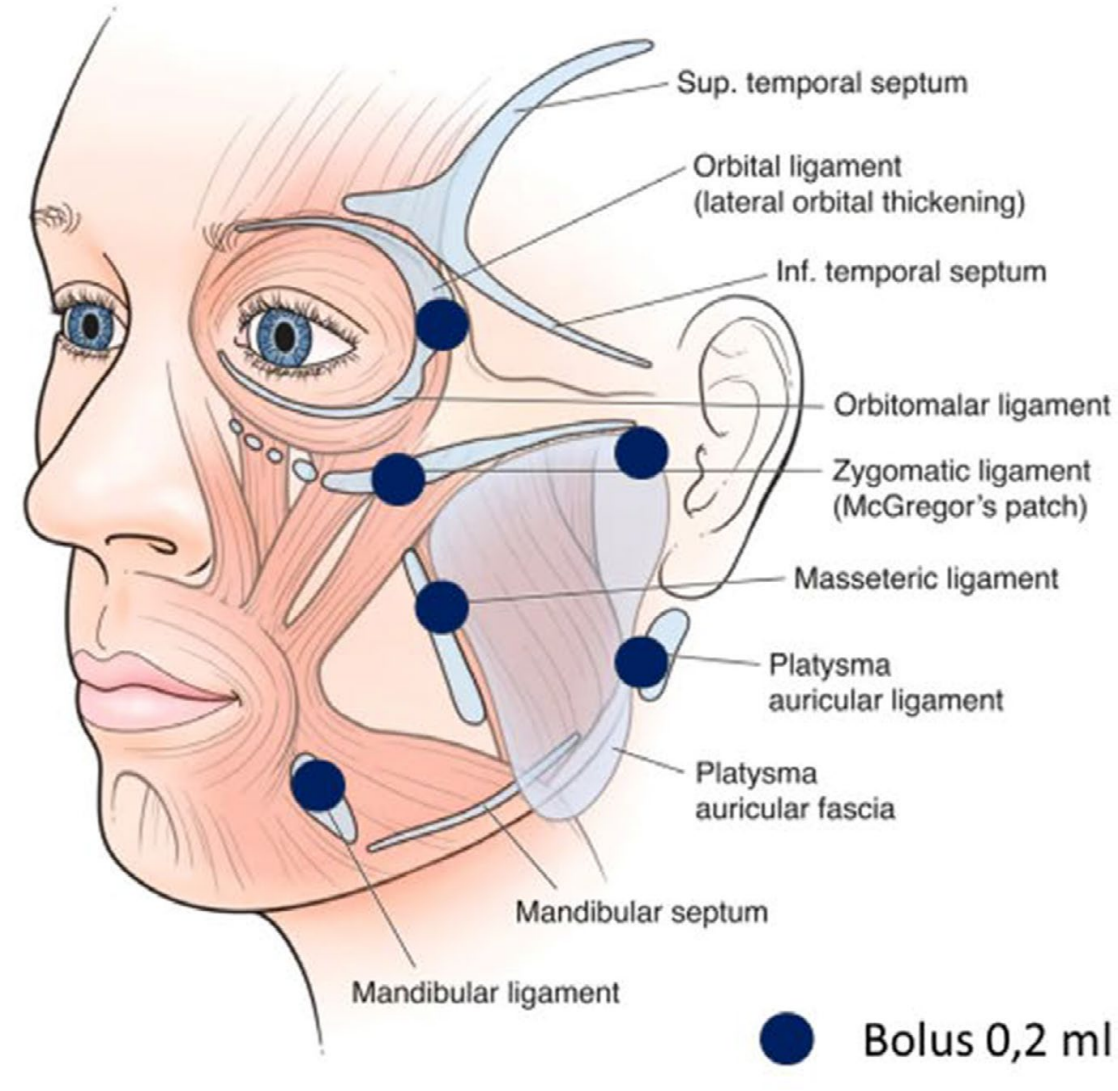
Injection protocol.

Measurements were performed at each injection point to obtain mean values (Figure 1).

### 2.3 Subjective Assessment

At T0, T1, and T2, each participant and investigator completed the Global Aesthetic Improvement Scale (GAIS), a 5‐point rating of perceived improvement in appearance compared to pre‐treatment conditions [9]. The GAIS questionnaire was used to assess the participants' satisfaction with the treatment results, which were scored on a range from −1 to 3, with −1 = worse than the baseline, 0 = no change, 1 = improved, 2 = much improved, and 3 = very much improved [7].

### 2.4 Treatment Procedure

The subjects received four sessions of monthly subdermal injections of 2.5 mL of the studied solution. No anesthetic cream was applied prior to the procedure. The injections were performed with a 27‐gauge, 13‐mm needle, applying subdermal 0.2 mL boluses using the ligament technique (Figure 1). Each bolus was injected at the point on the face corresponding to a specific ligament (lateral orbital thickening, platysma‐auricular fascia, zygomatic‐cutaneous ligament, upper masseteric ligament, or mandibular ligament).

### 2.5 Adverse Effect Assessment

Adverse effects, including pain, bruising, bleeding, lumps, skin irritation, redness, and a burning sensation at the site of injection, that occurred during and after treatment were noted.

### 2.6 Statistical Analysis

Descriptive statistics, such as mean, standard deviations, medians, and confidence intervals, were collected for all skin biophysical characteristics assessed (hydration, TEWL, elasticity, skin color, including melanin and erythema, skin thickness, and intensity) at each visit. The sign test and Wilcoxon test were used to compare the means between all visits. All statistical analyses were performed using STATISTICA 12 for Windows (StatSoft Inc.) Statistical significance for all analyses was set at *p* < 0.05.

## 3 Results

A total of 28 female volunteers aged 25–65 years (mean age, 43 years) participated in this study.

### 3.1 Subjective Measures

#### 3.1.1 GAIS Score

All participants noticed an improvement in appearance based on the GAIS score. The majority noticed significantly better appearance at T1 than at T0, with consistently high scores at T2. Only 22% of the participants experienced moderate improvement in appearance, and none of the participants showed worsening. The self‐reported GAIS scores improved from baseline to T2 (T0 score, 0; T1 score, 2.53; T2 score, 2.68). The investigator scores followed the same trend, with better scores at T1 than at T0 (T1 score, 2.45; T2 score, 2.74).

### 3.2 Adverse Effects

All participants completed the observational study. The injections showed beneficial effects on both participant satisfaction and skin biophysical parameters. The treatments were well tolerated by the participants, and there were minimal side effects. Three cases of superficial ecchymosis were recorded, and all these resolved spontaneously. During the injection procedure, there was minimal bleeding after skin puncture with a 29‐gauge needle, which stopped within 1–2 min in all cases. Some participants experienced mild pain during the injection, which spontaneously resided after the session. Application of the Jalupro bio‐cellulose mask helped relieve erythema and papules left at the injection points within 10–5 min of application after injection.

### 3.3 Objective Assessment—DermaLab Combo Tests

**FIGURE 2:**
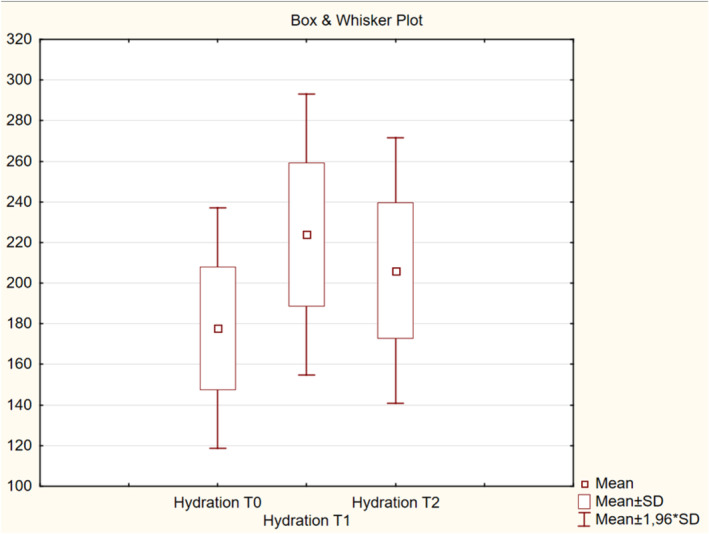
Change in skin hydration values from baseline.

**FIGURE 3:**
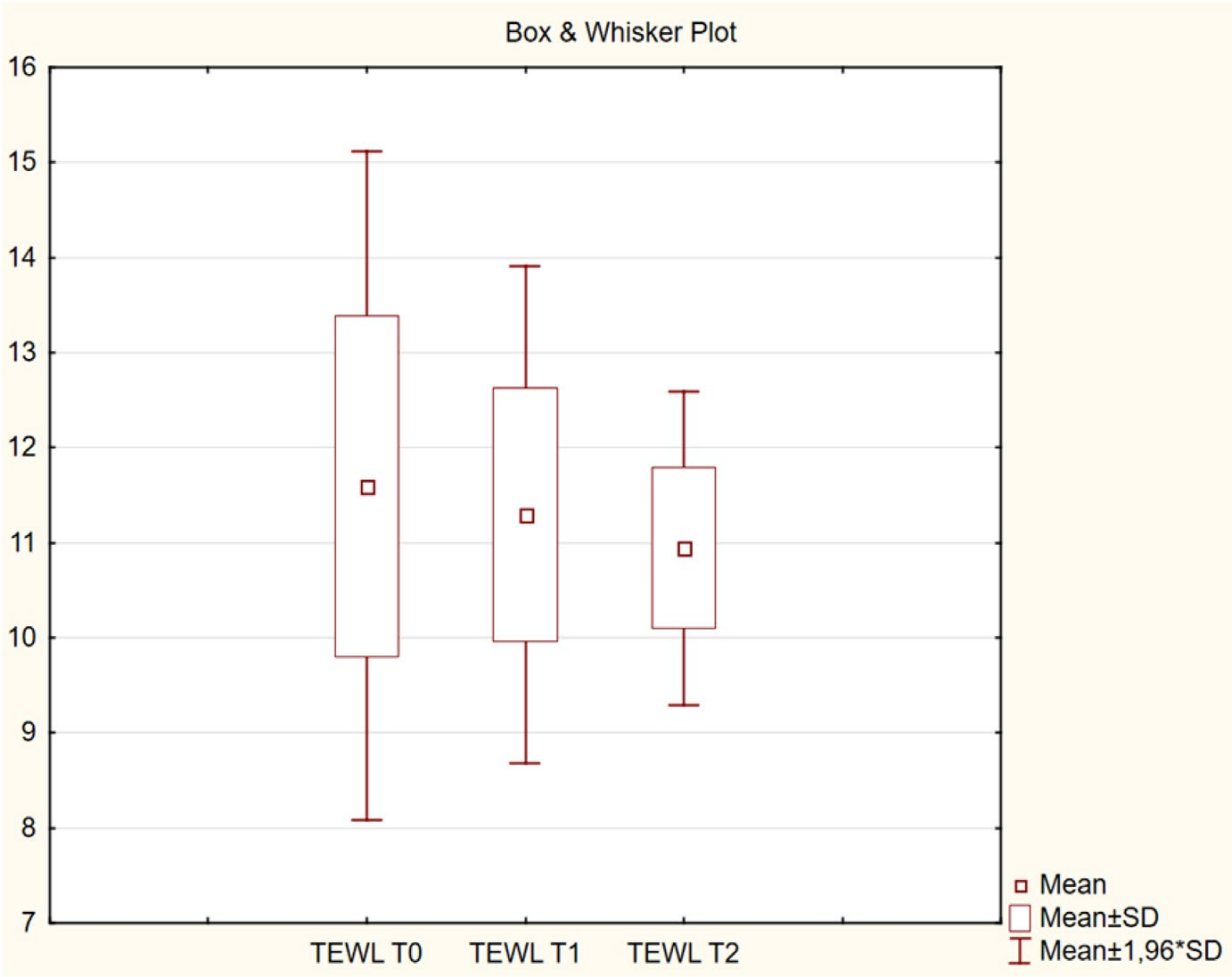
Change in trans‐epidermal water loss values from baseline.

**FIGURE 4:**
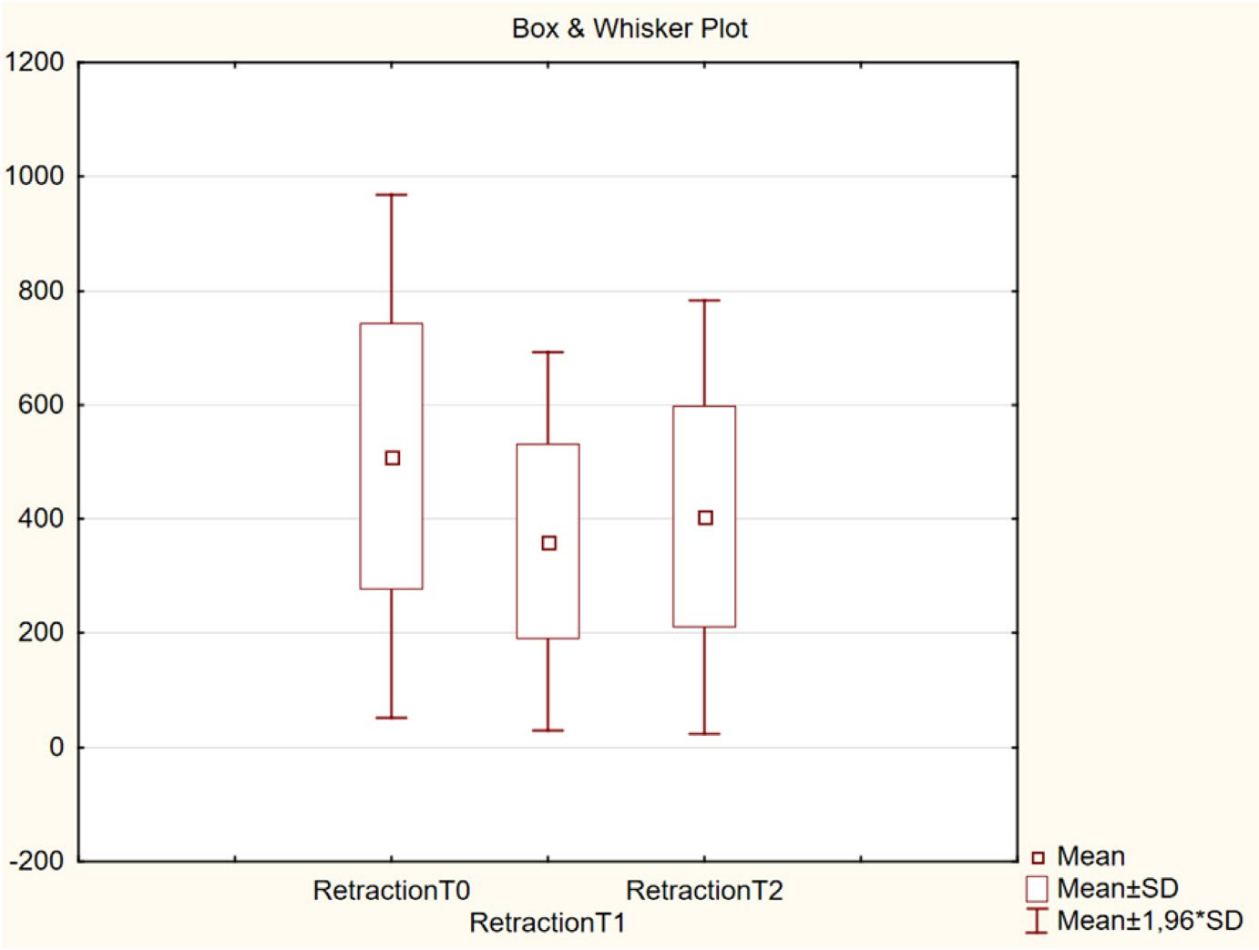
Change in elasticity values from baseline.

**FIGURE 5:**
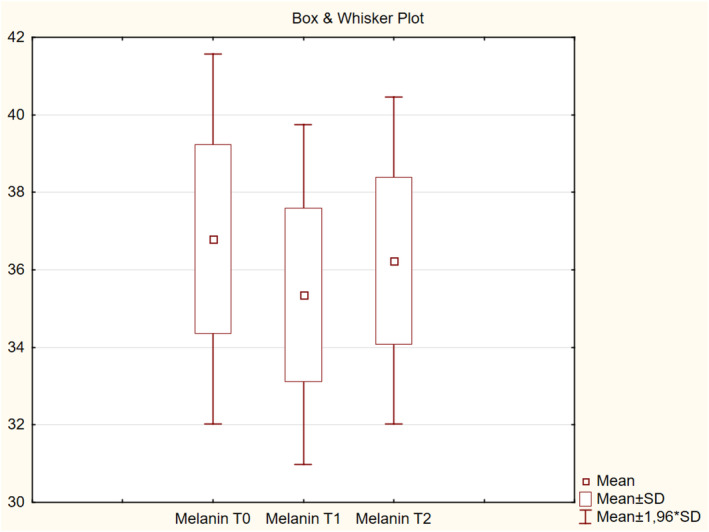
Change in melanin values from baseline.

**FIGURE 6:**
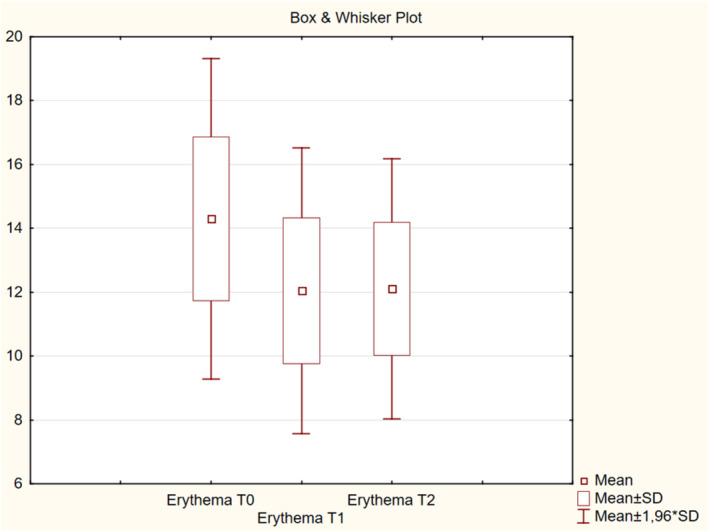
Change in erythema values from baseline.

**FIGURE 7:**
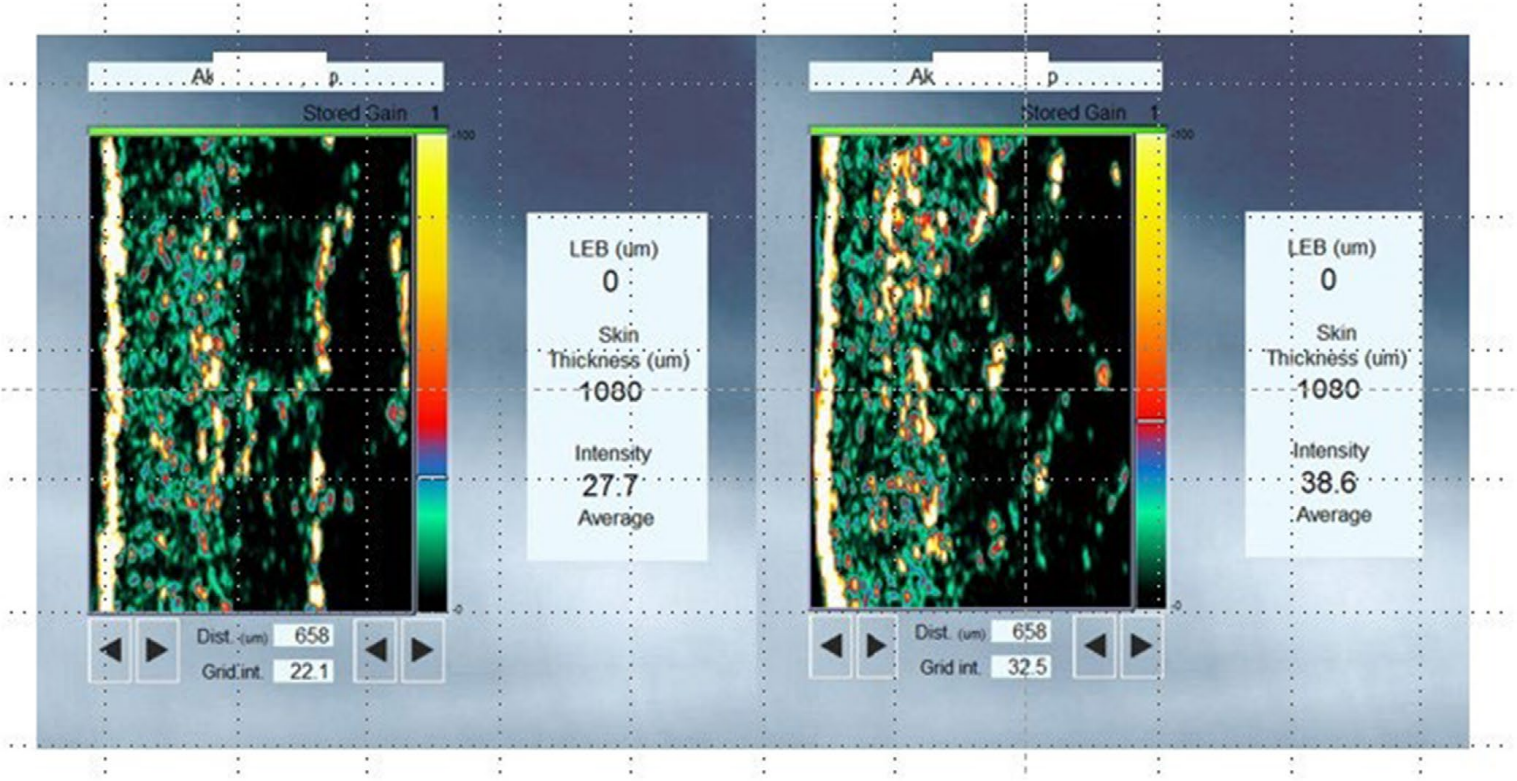
Improvement of intensity of collagen deposition score in dermis and RC assessed with high‐resolution ultrasound.

**FIGURE 8:**
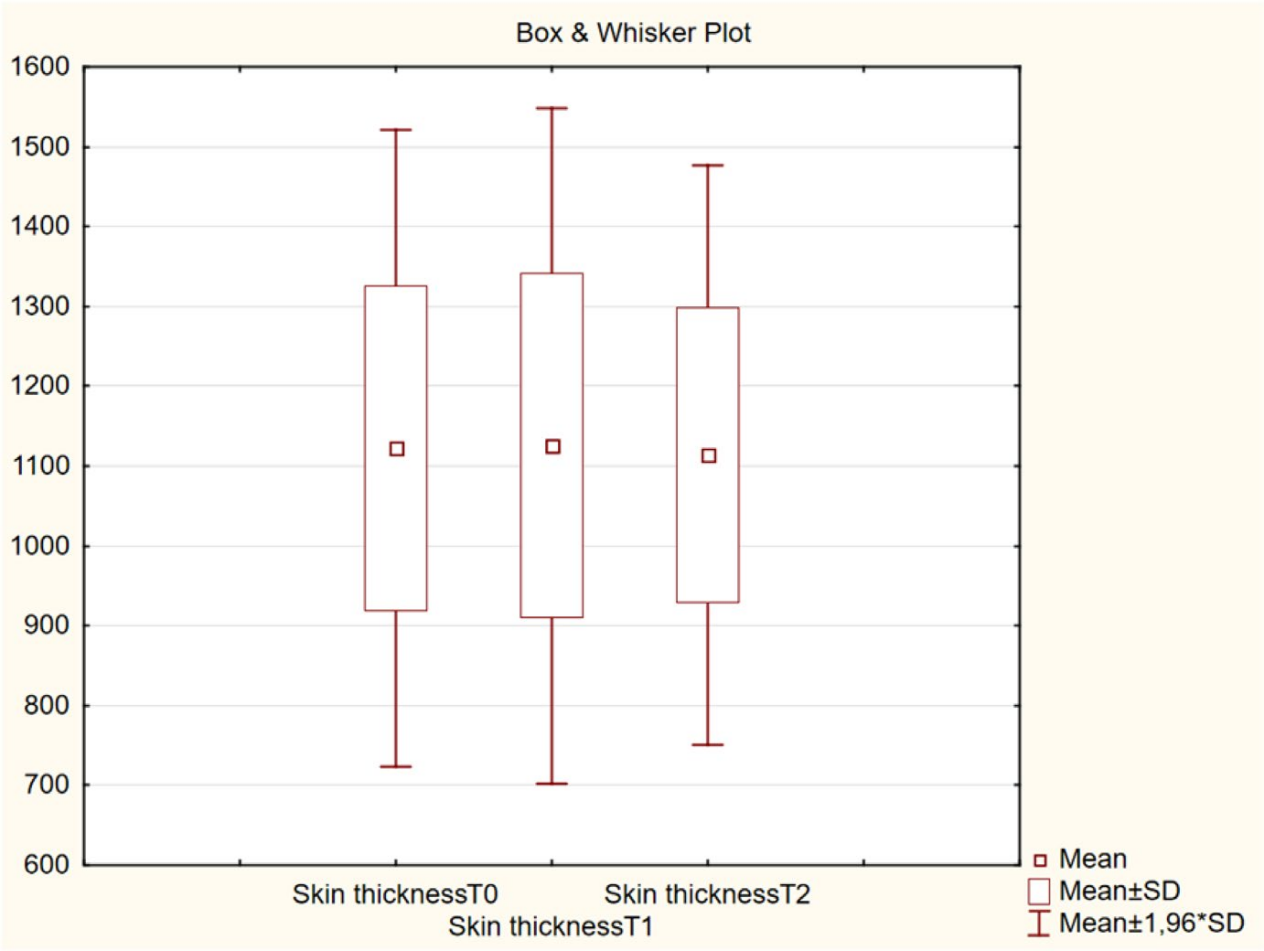
Change in skin thickness values from baseline.

**FIGURE 9:**
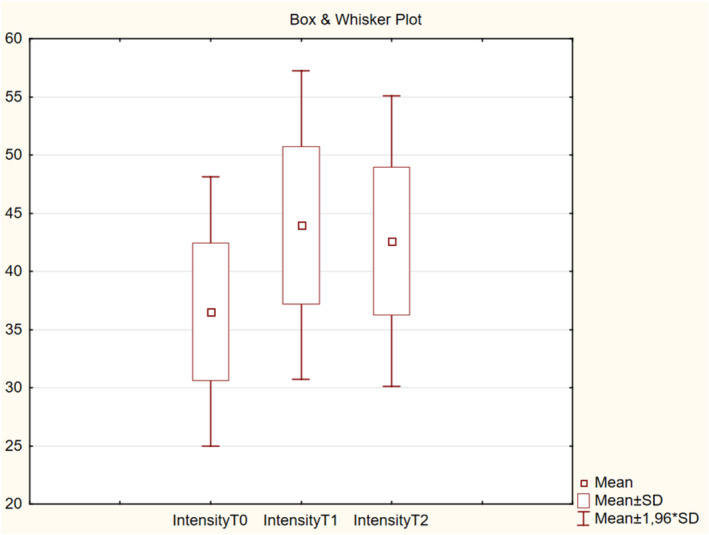
Change in ultrasound intensity values from baseline.

The results obtained from the derma laboratory tests, including for skin hydration, TEWL, melanin index, erythema index, elasticity, skin thickness, and intensity scores at T0, T1, and T2 are shown in Table 1 and Figures 2, 3, 4, 5, 6, 7, 8, 9. All parameters were checked bilaterally at each injection point and the average values at T0, T1, and T2 were calculated.

**TABLE 1:** Biophysical characteristics of the skin.

Parameter	Mean	Confidence −95%	Confidence 95%	Median	SD
Hydration					
Hydration at T0	177.83	166.13	189.52	181.00	30.16
Hydration at T1	223.96	210.28	237.65	230.50	35.28
Hydration at T2	206.20	193.28	219.13	210.00	33.33
TEWL					
TEWL at T0	11.60	10.90	12.29	11.68	1.79
TEWL at T1	11.29	10.78	11.81	10.95	1.34
TEWL at T2	10.95	10.62	11.27	10.90	0.84
Elasticity					
Retraction time at T0	510.04	419.50	600.57	438.50	233.49
Retraction time at T1	361.00	295.33	426.67	304.50	169.36
Retraction time at T2	404.18	329.04	479.31	334.00	193.77
Skin color—melanin					
Melanin at T0	36.80	35.85	37.74	36.60	2.43
Melanin at T1	35.36	34.49	36.23	35.35	2.24
Melanin at T2	36.24	35.40	37.07	36.30	2.15
Skin color—erythema					
Erythema at T0	14.30	13.31	15.30	14.15	2.56
Erythema at T1	12.05	11.16	12.94	12.10	2.29
Erythema at T2	12.11	11.30	12.91	12.10	2.08
Ultrasound—skin thickness					
Skin thickness at T0	1122.39	1043.52	1201.27	1120.50	203.42
Skin thickness at T1	1125.68	1042.01	1209.35	1118.50	215.77
Skin thickness at T2	1113.82	1041.96	1185.68	1120.00	185.32
Ultrasound—intensity					
Intensity at T0	36.53	34.24	38.82	36.55	5.91
Intensity at T1	43.97	41.34	46.59	43.15	6.77
Intensity at T2	42.60	40.13	45.07	40.90	6.37

### 3.4 Hydration (Moisture)

The hydration state was evaluated by measuring the conducting properties of the upper layers of the skin when subjected to an alternating voltage with a pin probe. The method is referred to as a conductance measurement and the output is presented in the unit of micro‐Siemens (μS).

The mean hydration level significantly increased from 177.83 μS at T0 to 223.96 at T1 (Table 1) (*p* < 0.001). Although the mean skin hydration level has decreased from 223.96 at T1 to 206.2 at T2 (*p* < 0.001), it was still higher than the baseline value (Figure 2).

### 3.5 Trans‐Epidermal Water Loss

Water loss was measured using a TEWL probe based on Nilsson's Vapor Pressure Gradient method, an open‐chamber method with minimal impact on the skin being examined and, accordingly, a very low reading bias. The TEWL probe measures the relative humidity and temperature inside the chamber and calculates the density gradient of water evaporation from the skin, which is converted into a TEWL value in g/m^2^/h.

The mean TEWL values were consistently below the 25 g/h/m^2^ critical threshold at T0, T1, and T2 (Table 1). No significant differences were found among visits (*p* > 0.05) (Figure 3).

### 3.6 Elasticity

The elasticity measurement was based on suction applied to the skin surface. The suction method features elevation and retraction phases. The retraction time was the time (in milliseconds) required for the skin to retract from its peak elevation to 33% of its peak elevation. This is indicated in the red section of the elasticity graph.

The mean retraction time decreased from 510 at T0 to 361 at T1 (*p* < 0.001) and to 404 at T2 (*p* < 0.001) (Figure 4).

### 3.7 Skin Color

The measurement of skin color, melanin (pigmentation), and erythema was based on an active color‐detecting chip, and illumination was provided by two high‐intensity white light‐emitting diodes. Melanin was used as a skin pigmentation index (Table 1), with higher values (measured in units) indicating greater pigment content in the skin (Figure 5). Erythema was defined as the redness index of the skin (Table 1). This measure was used to estimate the level of redness (hemoglobin) of the skin (Figure 6). There were no significant differences in skin color, melanin, or erythema among the visits (*p* > 0.05).

### 3.8 High‐Resolution Ultrasound Imaging

Ultrasound skin imaging detects the acoustic response of the skin and subcutaneous tissues when an acoustic pulse is transmitted and reflected from the skin. When the emitted acoustic pulse hits different skin structures, part of the pulse is reflected and transmitted further into the skin. The reflected signal is detected using an ultrasound transducer. After processing, the cross‐sectional image visualized on the screen represents the intensity (amplitude) analysis of the reflected signals (Figure 7).

In this study, we used a standard ultrasound probe that featured high‐resolution scanning and was specifically developed for skin imaging. It offered a good balance between resolution and penetration, with a scan length of 17.6 mm. This probe provided a circular scan movement with a medium‐sized footprint and a cross‐sectional image perpendicular to the skin surface. The measurement algorithm calculated skin thickness in micrometers, from which the average intensity (ultrasound reflection strength) was calculated in units. The intensity of the ultrasound passing through the dermis and subcutaneous layers is directly proportional to collagen deposition in the tissues, whereas the subcutaneous fat, muscle fibers, water, and blood return a low‐intensity signal.

There were no significant differences in skin thickness among the visits (T0, T1, and T2) (*p* > 0.05, Figure 8). Meanwhile, the mean intensity of collagen deposition in the dermis and subdermis increased from 36.5 at T0 to 43.9 at T1 (*p* < 0.001) and to 42.6 at T2 (*p* < 0.001) (Figure 9).

## 4 Discussion

In recent years, attention has shifted to more personalized, nonsurgical combination treatment plans that target the facial connective tissue and extracellular matrix. Connective tissue is a dynamic 3‐dimensional network of macromolecules, including collagens, elastin, and laminins that provide structural support to the skin and other facial structures [10]. Connective tissue is found not only in the dermis but also subdermally in the retinacular cutis and even deeper in the facial ligaments [11]. It progressively deteriorates with age, leading to facial ptosis, wrinkles, and loss of elasticity [5, 12, 13]. However, only a very limited range of products that target the improvement of collagen and elastin in the retinacular cutis and facial ligaments is available in the cosmetic market worldwide. Several studies have indicated that administering injections of non‐crosslinked HA supplemented with a cluster of AAs triggers chemotactic migration of fibroblasts to the treated site [14, 15]. This process leads to enhanced neocollagenesis, improved scarring, improved skin texture, accelerated wound healing, and reduced recovery time following invasive procedures [1, 14, 16].

In this study, based on the above theory and clinical practice, we developed an injection protocol corresponding to the anatomical location of facial ligaments to study the safety and efficacy of an injectable solution containing low‐ and high‐molecular‐weight HA, 7 AAs, and three biomimetic peptides. All participants were satisfied with the treatment, and there were no major side effects. For objective evaluations, significant improvements in hydration, elasticity, and collagen intensity scores were recorded. The mean hydration significantly increased by 25.9% (*p* < 0.001) at T1 from T0 and by 15.9% at T2 from T0. These measurements indicated that the study product effectively increased the hydration level of the skin in the short and long term. Further, the mean TEWL values at T0, T1, and T2 were always below the 25 g/h/m^2^ critical threshold. This proved that the studied product was safe and well tolerated and did not damage the stratum corneum's ability to retain moisture or effectively act as a barrier. Additionally, the slightly decreased TEWL scores after the course of treatment indicated that the product may increase the ability of the skin to retain moisture.

The mean retraction time decreased by 29.2% at T1 from T0 and by 20.7% at T2 from T0, indicating improved elasticity of the skin. There were no significant differences in skin color, both melanin and erythema, throughout the visits. This implied that the product did not promote vascular growth or cause post‐inflammatory hyperpigmentation, supporting that it can be safely injected according to the protocol described in this article. The mean intensity of collagen deposition in the dermal and subdermal layers of the skin was higher by 20.27% at T1 than that at T0 and by 16.71% at T2 than that at T0. Overall, the hydration level was improved from slightly dry to well moisturized after four injections in 25% of the participants. In addition, the level of collagen intensity in the dermis and subdermally improved from normal to high intensity, as detected by high‐resolution ultrasound, after the course of four injections in 42.9% of the participants.

The integrity and support of superficial and deep connective tissue play a pivotal role not only in maintaining structural stability, but also in contributing to clinical observations of facial shape. In this study, a lifting effect was also observed. The improvement of elasticity and collagen intensity score in the deep dermis and superficial subdermis and the improvement of facial shape and lifting, as observed in the before and after photos, point to the effectiveness of the product in supporting deep connective tissue. The injection technique aimed at the ligament appeared to provide support for lifting. The retinacular cutis is an extension of the facial retaining ligaments. Further studies are necessary to assess the lifting effect and potential strengthening of the facial retaining ligament.

The initial pilot study demonstrated promising safety and efficacy results, although it has some limitations, such as lack of prior research on the topic and small sample size. We are planning to continue the study with more participants from other centers. This expanded research aims to validate the initial findings, offering a more robust assessment of the injectable solution's potential for clinical application. Preliminary results indicate that the injectable solution is both safe and effective, with the extended study anticipated to further substantiate these findings and provide deeper insights into its mechanism of action and long‐term benefits.

## 5 Conclusion

Overall, the findings of this study support that a treatment regimen consisting of four injections of the studied product spaced 1 month apart is safe and well tolerated by subjects when injected subdermally according to the proposed technique. All participants experienced a significant improvement in skin hydration and elasticity. None of the participants experienced any deterioration. Jalupro Super Hydro provided a major hydrating effect, as evidenced by an increase in Corneometer scores, and improved skin elasticity and collagen deposition in dermis and subdermally, as evidenced by an increase in intensity score recorded with high‐resolution ultrasound imaging. Beneficial effects were still observed at the end of the study and at the visit 120 days after the last (4th) administration. These findings indicate that the mixture of high‐ and low‐molecular weight HA with seven AAs and three peptides can promote the assembly of extracellular matrix components and provide good hydration, antioxidant, and anti‐inflammatory tissue responses. The data on skin hydration, elasticity, and tension improvement are encouraging, especially considering the minimal invasiveness of the procedure and the limited number of sessions required.

### 5.1 Before/After Photos

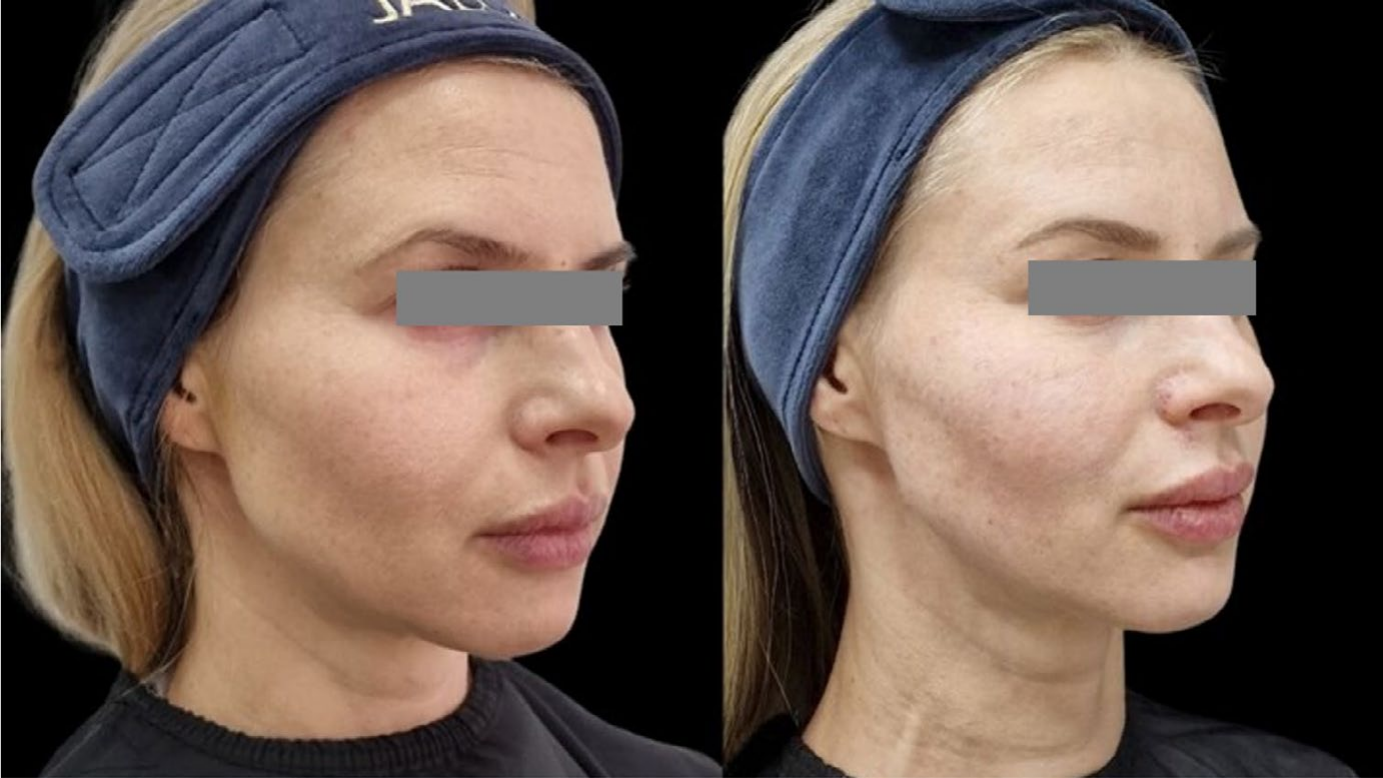

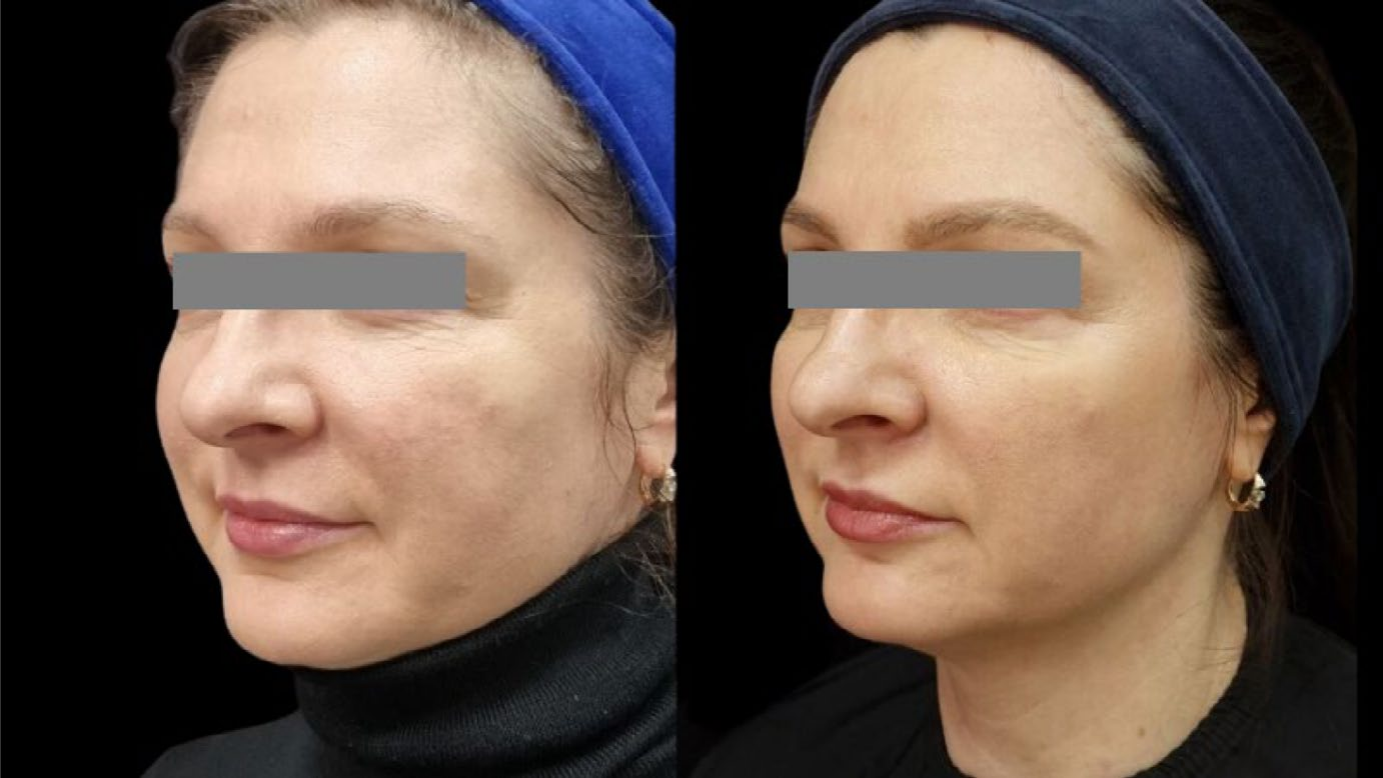

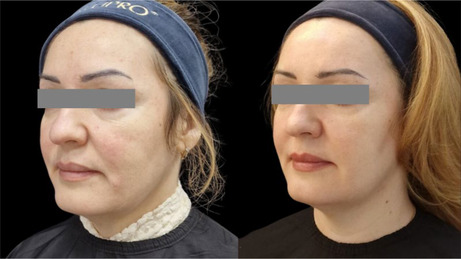

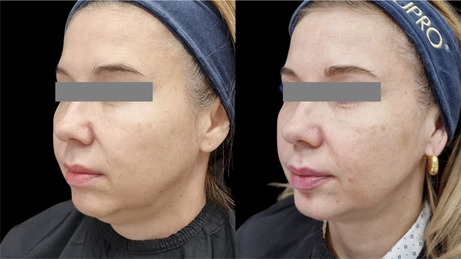

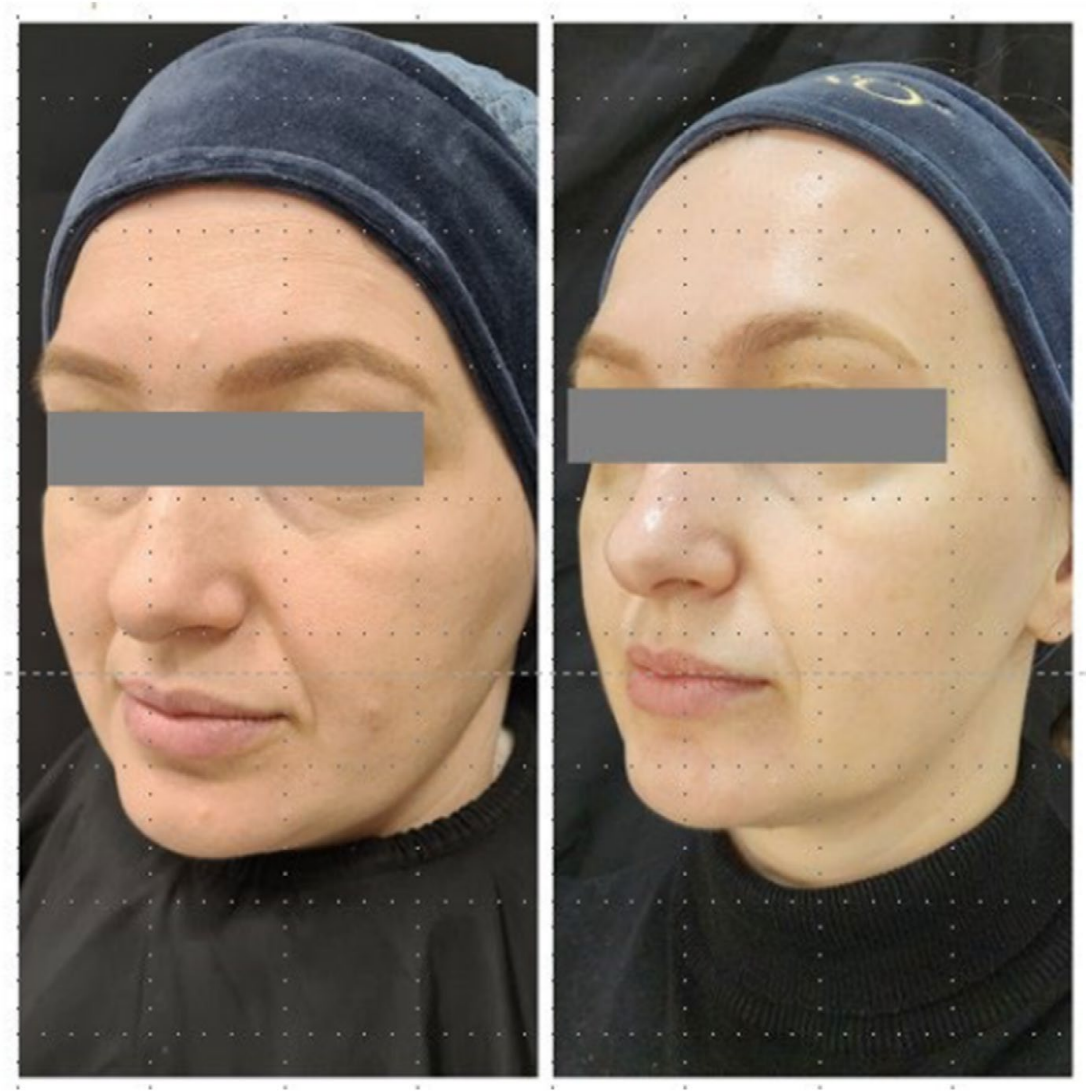

## Conflicts of Interest

The authors declare no conflicts of interest.

## Data Availability

The data that supports the findings of this study are available in the supplementary material of this article.
