# Correction to “Safety and Efficacy of an Injectable Solution Enriched with Sodium Hyaluronate, Amino Acids, and Peptides in Relation to Superficial Facial Connective Tissue”

**DOI:** 10.1111/jocd.70437

**Published:** 2025-09-19

Shelemba, E. , Olshanska, O. , Benoit, A.G. and Rumyantseva, E . Safety and Efficacy of an Injectable Solution Enriched With Sodium Hyaluronate, Amino Acids, and Peptides in Relation to Superficial Facial Connective Tissues (Dermis and Retinacular Cutis) Journal of Cosmetic Dermatology 2025 24 1 e16586. 10.1111/jocd.16586 39279301 PMC11743070

In the published version, the correction of two specific items that were inadvertently modified or misstated during the editing process:

1. **Gauge of the injection needle**

In the “Treatment Procedure” and “Adverse Effects” sections, the gauge of the needle used is stated incorrectly published. The correct values should be “27G” in the above sections.

2 **Terminology for hydration assessment**

In the “Conclusion” section, the term **“Corneometer”** was incorrectly published. The correct term should be **“corneometry”**.

We apologize for these errors.

